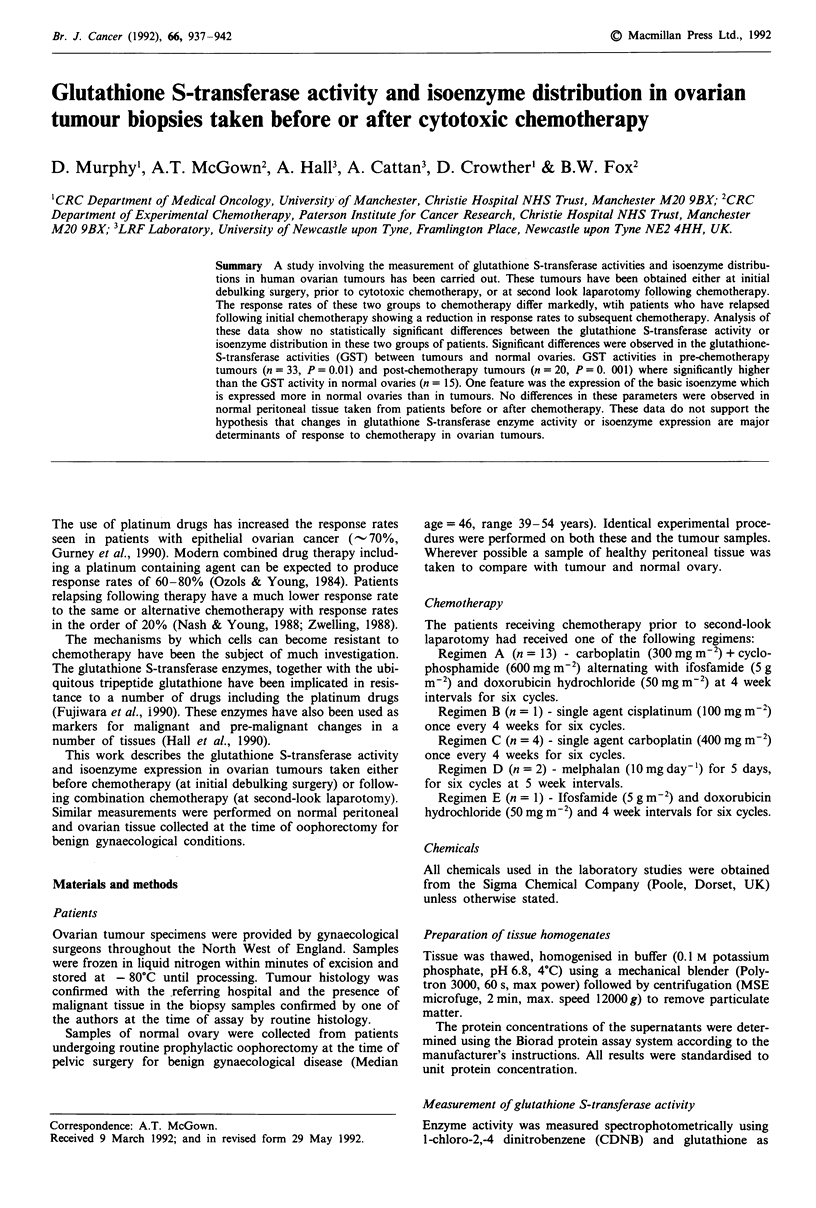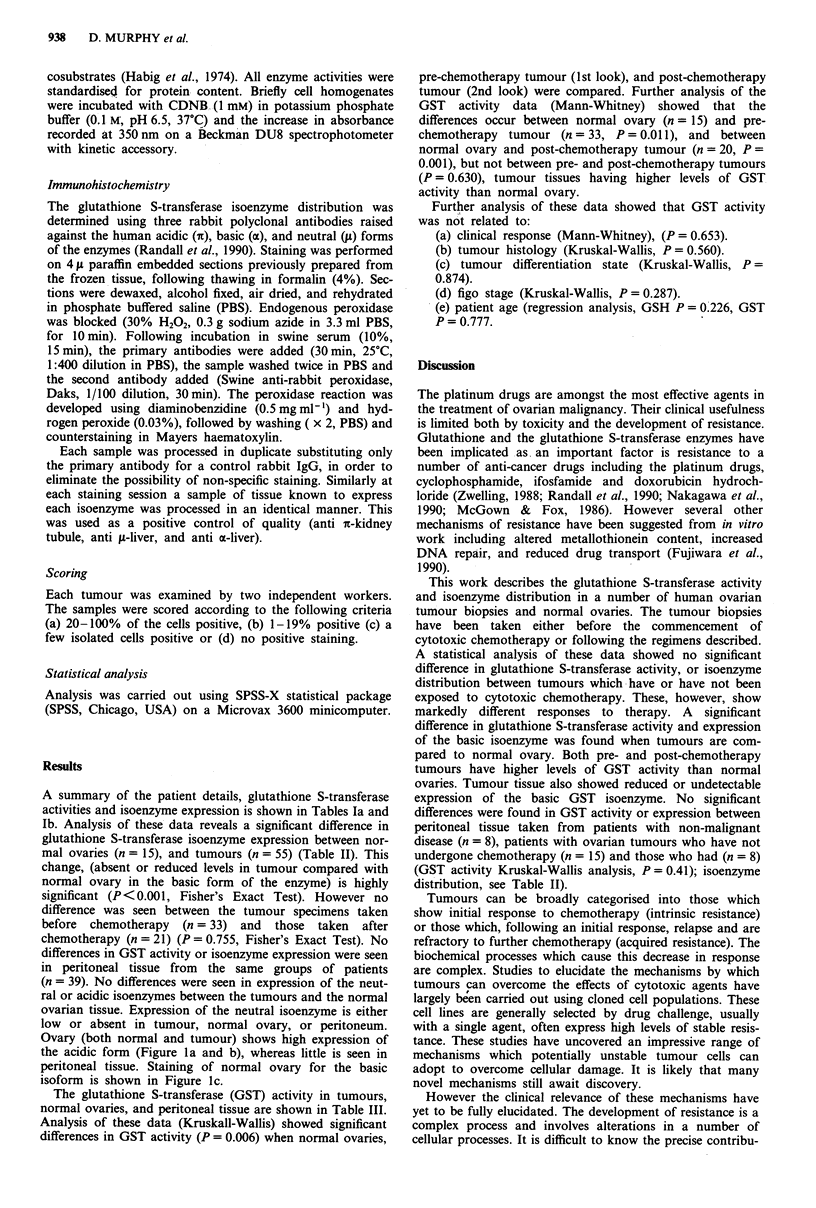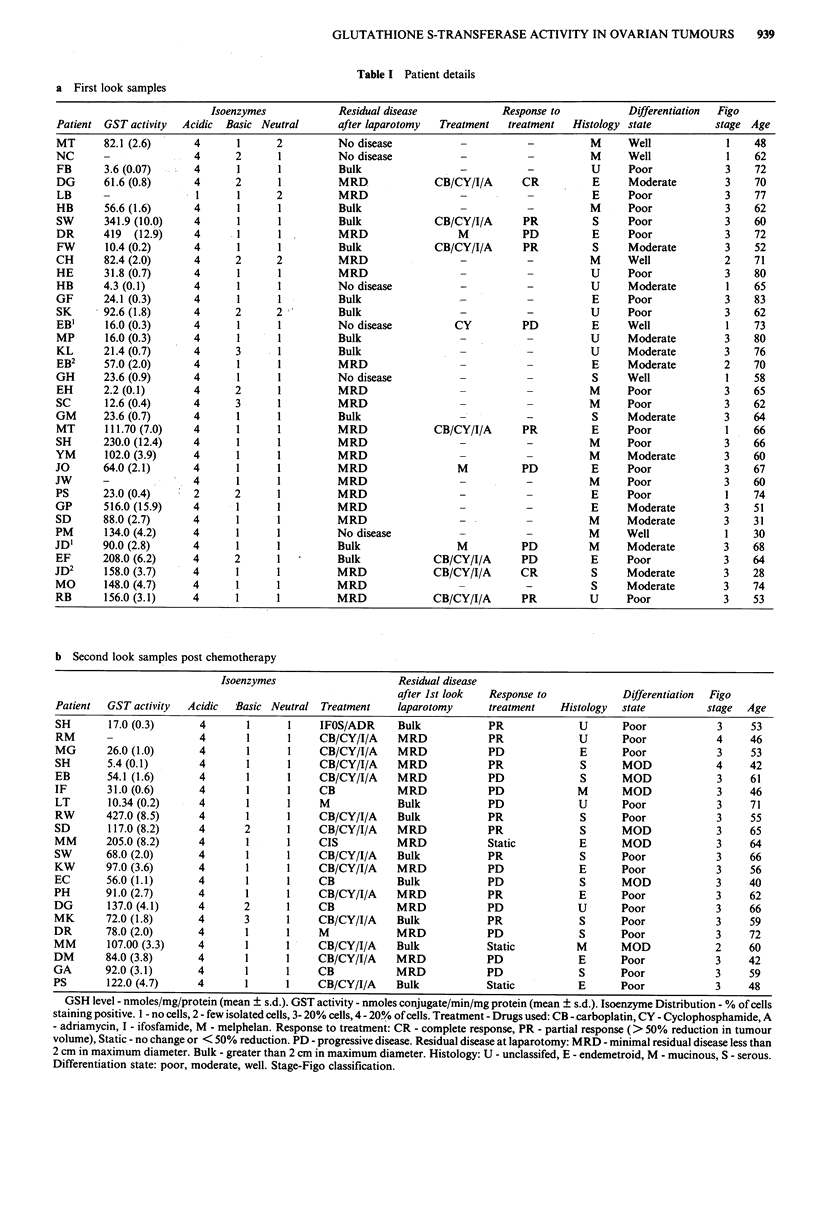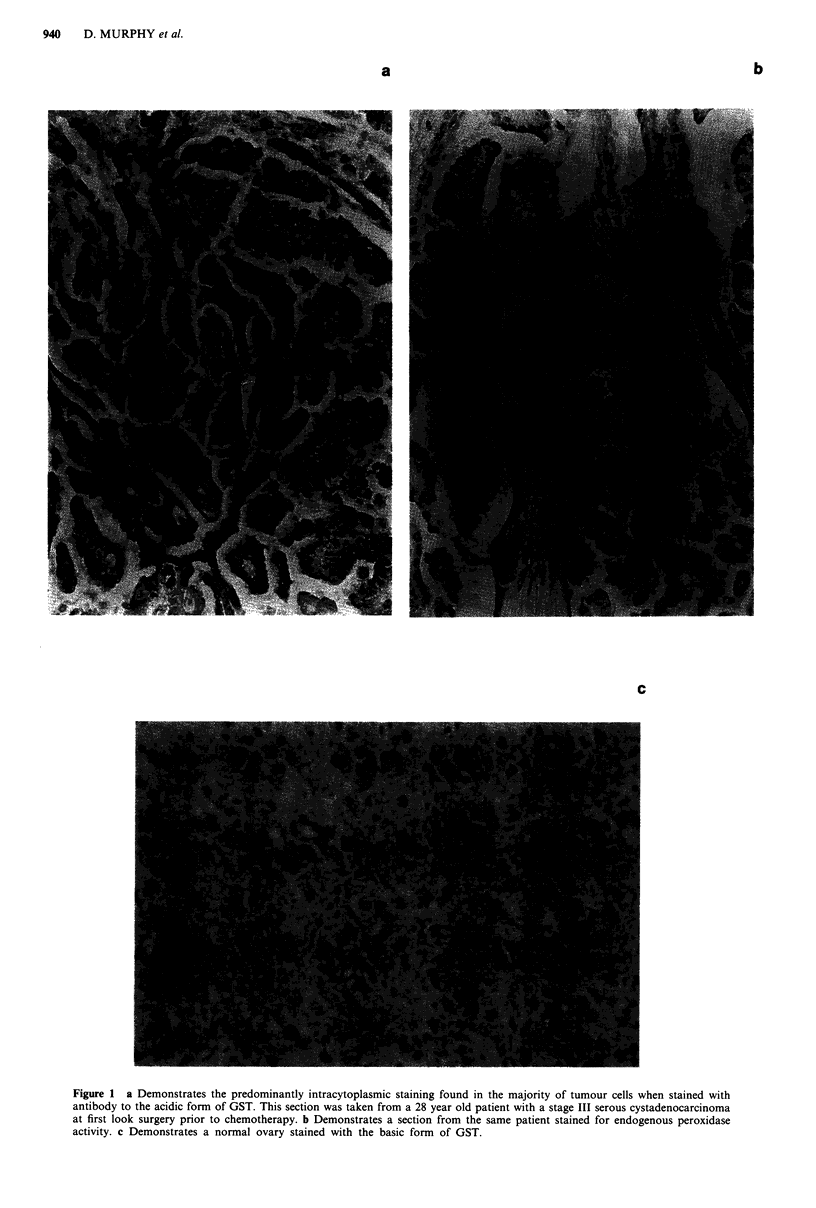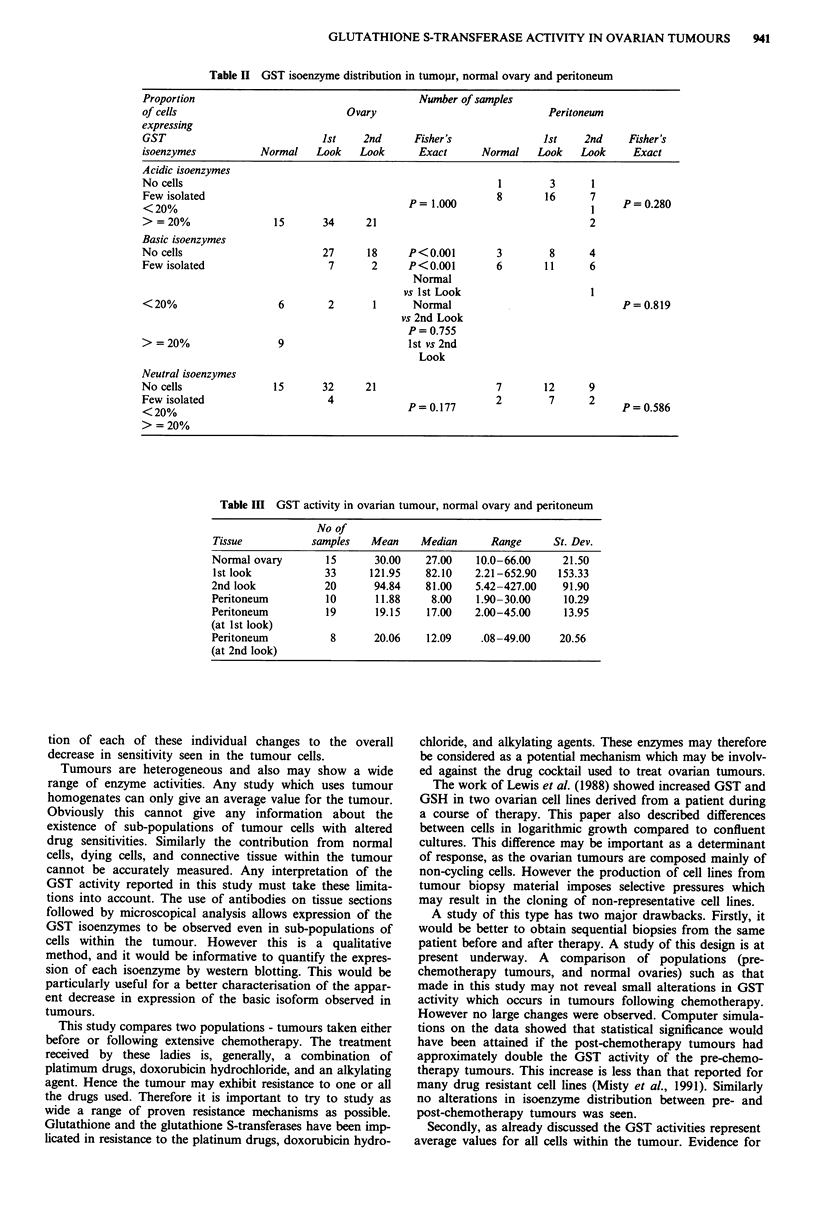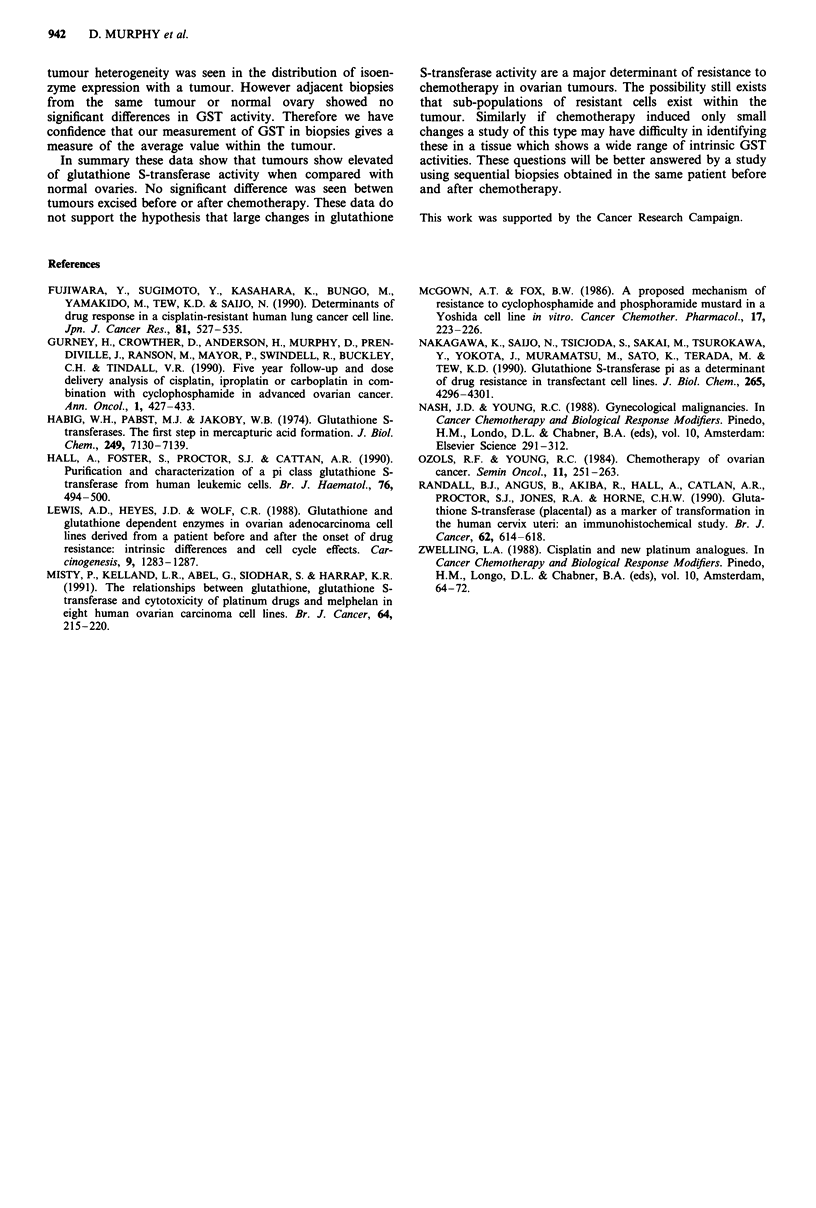# Glutathione S-transferase activity and isoenzyme distribution in ovarian tumour biopsies taken before or after cytotoxic chemotherapy.

**DOI:** 10.1038/bjc.1992.389

**Published:** 1992-11

**Authors:** D. Murphy, A. T. McGown, A. Hall, A. Cattan, D. Crowther, B. W. Fox

**Affiliations:** CRC Department of Medical Oncology, University of Manchester, Christie Hospital NHS Trust, UK.

## Abstract

**Images:**


Br. J. Cancer (1992), 66, 937-942 © Macmillan Press Ltd., 1992

### Glutathione S-transferase activity and isoenzyme distribution in ovarian tumour biopsies taken before or after cytotoxic chemotherapy

D. Murphy[1], A.T. McGown[2], A. Hall[3], A. Cattan[3], D. Crowther[1] & B.W. Fox[2]

[1]*CRC Department of Medical Oncology, University of Manchester, Christie Hospital NHS Trust, Manchester M20 9BX;* [2]*CRC Department of Experimental Chemotherapy, Paterson Institute for Cancer Research, Christie Hospital NHS Trust, Manchester M20 9BX;* [3]*LRF Laboratory, University of Newcastle upon Tyne, Framlington Place, Newcastle upon Tyne NE2 4HH, UK.*

**Summary** A study involving the measurement of glutathione S-transferase activities and isoenzyme distributions in human ovarian tumours has been carried out. These tumours have been obtained either at initial debulking surgery, prior to cytotoxic chemotherapy, or at second look laparotomy following chemotherapy. The response rates of these two groups to chemotherapy differ markedly, wtih patients who have relapsed following initial chemotherapy showing a reduction in response rates to subsequent chemotherapy. Analysis of these data show no statistically significant differences between the glutathione S-transferase activity or isoenzyme distribution in these two groups of patients. Significant differences were observed in the glutathione-S-transferase activities (GST) between tumours and normal ovaries. GST activities in pre-chemotherapy tumours ($n = 33$, $P = 0.01$) and post-chemotherapy tumours ($n = 20$, $P = 0.$ 001) where significantly higher than the GST activity in normal ovaries ($n = 15$). One feature was the expression of the basic isoenzyme which is expressed more in normal ovaries than in tumours. No differences in these parameters were observed in normal peritoneal tissue taken from patients before or after chemotherapy. These data do not support the hypothesis that changes in glutathione S-transferase enzyme activity or isoenzyme expression are major determinants of response to chemotherapy in ovarian tumours.

The use of platinum drugs has increased the response rates seen in patients with epithelial ovarian cancer (~70%, Gurney *et al.*, 1990). Modern combined drug therapy including a platinum containing agent can be expected to produce response rates of 60-80% (Ozols & Young, 1984). Patients relapsing following therapy have a much lower response rate to the same or alternative chemotherapy with response rates in the order of 20% (Nash & Young, 1988; Zwelling, 1988).

The mechanisms by which cells can become resistant to chemotherapy have been the subject of much investigation. The glutathione S-transferase enzymes, together with the ubiquitous tripeptide glutathione have been implicated in resistance to a number of drugs including the platinum drugs (Fujiwara *et al.*, 1990). These enzymes have also been used as markers for malignant and pre-malignant changes in a number of tissues (Hall *et al.*, 1990).

This work describes the glutathione S-transferase activity and isoenzyme expression in ovarian tumours taken either before chemotherapy (at initial debulking surgery) or following combination chemotherapy (at second-look laparotomy). Similar measurements were performed on normal peritoneal and ovarian tissue collected at the time of oophorectomy for benign gynaecological conditions.

### Materials and methods

### *Patients*

Ovarian tumour specimens were provided by gynaecological surgeons throughout the North West of England. Samples were frozen in liquid nitrogen within minutes of excision and stored at − 80°C until processing. Tumour histology was confirmed with the referring hospital and the presence of malignant tissue in the biopsy samples confirmed by one of the authors at the time of assay by routine histology.

Samples of normal ovary were collected from patients undergoing routine prophylactic oophorectomy at the time of pelvic surgery for benign gynaecological disease (Median age = 46, range 39-54 years). Identical experimental procedures were performed on both these and the tumour samples. Wherever possible a sample of healthy peritoneal tissue was taken to compare with tumour and normal ovary.

### *Chemotherapy*

The patients receiving chemotherapy prior to second-look laparotomy had received one of the following regimens:

Regimen A ($n = 13$) - carboplatin (300 mg m$^{-2}$) + cyclophosphamide (600 mg m$^{-2}$) alternating with ifosfamide (5 g m$^{-2}$) and doxorubicin hydrochloride (50 mg m$^{-2}$) at 4 week intervals for six cycles.

Regimen B ($n = 1$) - single agent cisplatinum (100 mg m$^{-2}$) once every 4 weeks for six cycles.

Regimen C ($n = 4$) - single agent carboplatin (400 mg m$^{-2}$) once every 4 weeks for six cycles.

Regimen D ($n = 2$) - melphalan (10 mg day$^{-1}$) for 5 days, for six cycles at 5 week intervals.

Regimen E ($n = 1$) - Ifosfamide (5 g m$^{-2}$) and doxorubicin hydrochloride (50 mg m$^{-2}$) and 4 week intervals for six cycles.

### *Chemicals*

All chemicals used in the laboratory studies were obtained from the Sigma Chemical Company (Poole, Dorset, UK) unless otherwise stated.

### *Preparation of tissue homogenates*

Tissue was thawed, homogenised in buffer (0.1 M potassium phosphate, pH 6.8, 4°C) using a mechanical blender (Polytron 3000, 60 s, max power) followed by centrifugation (MSE microfuge, 2 min, max. speed 12000 *g*) to remove particulate matter.

The protein concentrations of the supernatants were determined using the Biorad protein assay system according to the manufacturer's instructions. All results were standardised to unit protein concentration.

### *Measurement of glutathione S-transferase activity*

Enzyme activity was measured spectrophotometrically using 1-chloro-2,-4 dinitrobenzene (CDNB) and glutathione as

Correspondence: A.T. McGown.
Received 9 March 1992; and in revised form 29 May 1992.

cosubstrates (Habig et al., 1974). All enzyme activities were standardised for protein content. Briefly cell homogenates were incubated with CDNB (1 mM) in potassium phosphate buffer (0.1 M, pH 6.5, 37°C) and the increase in absorbance recorded at 350 nm on a Beckman DU8 spectrophotometer with kinetic accessory.

*Immunohistochemistry*

The glutathione S-transferase isoenzyme distribution was determined using three rabbit polyclonal antibodies raised against the human acidic (π), basic (α), and neutral (μ) forms of the enzymes (Randall et al., 1990). Staining was performed on 4 μ paraffin embedded sections previously prepared from the frozen tissue, following thawing in formalin (4%). Sections were dewaxed, alcohol fixed, air dried, and rehydrated in phosphate buffered saline (PBS). Endogenous peroxidase was blocked (30% $H_2O_2$, 0.3 g sodium azide in 3.3 ml PBS, for 10 min). Following incubation in swine serum (10%, 15 min), the primary antibodies were added (30 min, 25°C, 1:400 dilution in PBS), the sample washed twice in PBS and the second antibody added (Swine anti-rabbit peroxidase, Daks, 1/100 dilution, 30 min). The peroxidase reaction was developed using diaminobenzidine (0.5 mg $ml^{-1}$) and hydrogen peroxide (0.03%), followed by washing ( × 2, PBS) and counterstaining in Mayers haematoxylin.

Each sample was processed in duplicate substituting only the primary antibody for a control rabbit IgG, in order to eliminate the possibility of non-specific staining. Similarly at each staining session a sample of tissue known to express each isoenzyme was processed in an identical manner. This was used as a positive control of quality (anti π-kidney tubule, anti μ-liver, and anti α-liver).

*Scoring*

Each tumour was examined by two independent workers. The samples were scored according to the following criteria (a) 20–100% of the cells positive, (b) 1–19% positive (c) a few isolated cells positive or (d) no positive staining.

*Statistical analysis*

Analysis was carried out using SPSS-X statistical package (SPSS, Chicago, USA) on a Microvax 3600 minicomputer.

### Results

A summary of the patient details, glutathione S-transferase activities and isoenzyme expression is shown in Tables Ia and Ib. Analysis of these data reveals a significant difference in glutathione S-transferase isoenzyme expression between normal ovaries ($n = 15$), and tumours ($n = 55$) (Table II). This change, (absent or reduced levels in tumour compared with normal ovary in the basic form of the enzyme) is highly significant ($P < 0.001$, Fisher's Exact Test). However no difference was seen between the tumour specimens taken before chemotherapy ($n = 33$) and those taken after chemotherapy ($n = 21$) ($P = 0.755$, Fisher's Exact Test). No differences in GST activity or isoenzyme expression were seen in peritoneal tissue from the same groups of patients ($n = 39$). No differences were seen in expression of the neutral or acidic isoenzymes between the tumours and the normal ovarian tissue. Expression of the neutral isoenzyme is either low or absent in tumour, normal ovary, or peritoneum. Ovary (both normal and tumour) shows high expression of the acidic form (Figure 1a and b), whereas little is seen in peritoneal tissue. Staining of normal ovary for the basic isoform is shown in Figure 1c.

The glutathione S-transferase (GST) activity in tumours, normal ovaries, and peritoneal tissue are shown in Table III. Analysis of these data (Kruskall-Wallis) showed significant differences in GST activity ($P = 0.006$) when normal ovaries, pre-chemotherapy tumour (1st look), and post-chemotherapy tumour (2nd look) were compared. Further analysis of the GST activity data (Mann-Whitney) showed that the differences occur between normal ovary ($n = 15$) and pre-chemotherapy tumour ($n = 33$, $P = 0.011$), and between normal ovary and post-chemotherapy tumour ($n = 20$, $P = 0.001$), but not between pre- and post-chemotherapy tumours ($P = 0.630$), tumour tissues having higher levels of GST activity than normal ovary.

Further analysis of these data showed that GST activity was not related to:

(a) clinical response (Mann-Whitney), ($P = 0.653$).
(b) tumour histology (Kruskal-Wallis, $P = 0.560$).
(c) tumour differentiation state (Kruskal-Wallis, $P = 0.874$).
(d) figo stage (Kruskal-Wallis, $P = 0.287$).
(e) patient age (regression analysis, GSH $P = 0.226$, GST $P = 0.777$.

### Discussion

The platinum drugs are amongst the most effective agents in the treatment of ovarian malignancy. Their clinical usefulness is limited both by toxicity and the development of resistance. Glutathione and the glutathione S-transferase enzymes have been implicated as an important factor is resistance to a number of anti-cancer drugs including the platinum drugs, cyclophosphamide, ifosfamide and doxorubicin hydrochloride (Zwelling, 1988; Randall et al., 1990; Nakagawa et al., 1990; McGown & Fox, 1986). However several other mechanisms of resistance have been suggested from *in vitro* work including altered metallothionein content, increased DNA repair, and reduced drug transport (Fujiwara et al., 1990).

This work describes the glutathione S-transferase activity and isoenzyme distribution in a number of human ovarian tumour biopsies and normal ovaries. The tumour biopsies have been taken either before the commencement of cytotoxic chemotherapy or following the regimens described. A statistical analysis of these data showed no significant difference in glutathione S-transferase activity, or isoenzyme distribution between tumours which have or have not been exposed to cytotoxic chemotherapy. These, however, show markedly different responses to therapy. A significant difference in glutathione S-transferase activity and expression of the basic isoenzyme was found when tumours are compared to normal ovary. Both pre- and post-chemotherapy tumours have higher levels of GST activity than normal ovaries. Tumour tissue also showed reduced or undetectable expression of the basic GST isoenzyme. No significant differences were found in GST activity or expression between peritoneal tissue taken from patients with non-malignant disease ($n = 8$), patients with ovarian tumours who have not undergone chemotherapy ($n = 15$) and those who had ($n = 8$) (GST activity Kruskal-Wallis analysis, $P = 0.41$); isoenzyme distribution, see Table II).

Tumours can be broadly categorised into those which show initial response to chemotherapy (intrinsic resistance) or those which, following an initial response, relapse and are refractory to further chemotherapy (acquired resistance). The biochemical processes which cause this decrease in response are complex. Studies to elucidate the mechanisms by which tumours can overcome the effects of cytotoxic agents have largely been carried out using cloned cell populations. These cell lines are generally selected by drug challenge, usually with a single agent, often express high levels of stable resistance. These studies have uncovered an impressive range of mechanisms which potentially unstable tumour cells can adopt to overcome cellular damage. It is likely that many novel mechanisms still await discovery.

However the clinical relevance of these mechanisms have yet to be fully elucidated. The development of resistance is a complex process and involves alterations in a number of cellular processes. It is difficult to know the precise contribu-

**Table I** Patient details

**a** First look samples

| Patient | GST activity | Isoenzymes Acidic | Isoenzymes Basic | Isoenzymes Neutral | Residual disease after laparotomy | Treatment | Response to treatment | Histology | Differentiation state | Figo stage | Age |
|---|---|---|---|---|---|---|---|---|---|---|---|
| MT | 82.1 (2.6) | 4 | 1 | 2 | No disease | – | – | M | Well | 1 | 48 |
| NC | – | 4 | 2 | 1 | No disease | – | – | M | Well | 1 | 62 |
| FB | 3.6 (0.07) | 4 | 1 | 1 | Bulk | – | – | U | Poor | 3 | 72 |
| DG | 61.6 (0.8) | 4 | 2 | 1 | MRD | CB/CY/I/A | CR | E | Moderate | 3 | 70 |
| LB | – | 1 | 1 | 2 | MRD | – | – | E | Poor | 3 | 77 |
| HB | 56.6 (1.6) | 4 | 1 | 1 | Bulk | – | – | M | Poor | 3 | 62 |
| SW | 341.9 (10.0) | 4 | 1 | 1 | Bulk | CB/CY/I/A | PR | S | Poor | 3 | 60 |
| DR | 419 (12.9) | 4 | 1 | 1 | MRD | M | PD | E | Poor | 3 | 72 |
| FW | 10.4 (0.2) | 4 | 1 | 1 | Bulk | CB/CY/I/A | PR | S | Moderate | 3 | 52 |
| CH | 82.4 (2.0) | 4 | 2 | 2 | MRD | – | – | M | Well | 2 | 71 |
| HE | 31.8 (0.7) | 4 | 1 | 1 | MRD | – | – | U | Poor | 3 | 80 |
| HB | 4.3 (0.1) | 4 | 1 | 1 | No disease | – | – | U | Moderate | 1 | 65 |
| GF | 24.1 (0.3) | 4 | 1 | 1 | Bulk | – | – | E | Poor | 3 | 83 |
| SK | 92.6 (1.8) | 4 | 2 | 2 | Bulk | – | – | U | Poor | 3 | 62 |
| EB[1] | 16.0 (0.3) | 4 | 1 | 1 | No disease | CY | PD | E | Well | 1 | 73 |
| MP | 16.0 (0.3) | 4 | 1 | 1 | Bulk | – | – | U | Moderate | 3 | 80 |
| KL | 21.4 (0.7) | 4 | 3 | 1 | Bulk | – | – | U | Moderate | 3 | 76 |
| EB[2] | 57.0 (2.0) | 4 | 1 | 1 | MRD | – | – | E | Moderate | 2 | 70 |
| GH | 23.6 (0.9) | 4 | 1 | 1 | No disease | – | – | S | Well | 1 | 58 |
| EH | 2.2 (0.1) | 4 | 2 | 1 | MRD | – | – | M | Poor | 3 | 65 |
| SC | 12.6 (0.4) | 4 | 3 | 1 | MRD | – | – | M | Poor | 3 | 62 |
| GM | 23.6 (0.7) | 4 | 1 | 1 | Bulk | – | – | S | Moderate | 3 | 64 |
| MT | 111.70 (7.0) | 4 | 1 | 1 | MRD | CB/CY/I/A | PR | E | Poor | 1 | 66 |
| SH | 230.0 (12.4) | 4 | 1 | 1 | MRD | – | – | M | Poor | 3 | 66 |
| YM | 102.0 (3.9) | 4 | 1 | 1 | MRD | – | – | M | Moderate | 3 | 60 |
| JO | 64.0 (2.1) | 4 | 1 | 1 | MRD | M | PD | E | Poor | 3 | 67 |
| JW | – | 4 | 1 | 1 | MRD | – | – | M | Poor | 3 | 60 |
| PS | 23.0 (0.4) | 2 | 2 | 1 | MRD | – | – | E | Poor | 1 | 74 |
| GP | 516.0 (15.9) | 4 | 1 | 1 | MRD | – | – | E | Moderate | 3 | 51 |
| SD | 88.0 (2.7) | 4 | 1 | 1 | MRD | – | – | M | Moderate | 3 | 31 |
| PM | 134.0 (4.2) | 4 | 1 | 1 | No disease | – | – | M | Well | 1 | 30 |
| JD[1] | 90.0 (2.8) | 4 | 1 | 1 | Bulk | M | PD | M | Moderate | 3 | 68 |
| EF | 208.0 (6.2) | 4 | 2 | 1 | Bulk | CB/CY/I/A | PD | E | Poor | 3 | 64 |
| JD[2] | 158.0 (3.7) | 4 | 1 | 1 | MRD | CB/CY/I/A | CR | S | Moderate | 3 | 28 |
| MO | 148.0 (4.7) | 4 | 1 | 1 | MRD | – | – | S | Moderate | 3 | 74 |
| RB | 156.0 (3.1) | 4 | 1 | 1 | MRD | CB/CY/I/A | PR | U | Poor | 3 | 53 |

**b** Second look samples post chemotherapy

| Patient | GST activity | Isoenzymes Acidic | Isoenzymes Basic | Isoenzymes Neutral | Treatment | Residual disease after 1st look laparotomy | Response to treatment | Histology | Differentiation state | Figo stage | Age |
|---|---|---|---|---|---|---|---|---|---|---|---|
| SH | 17.0 (0.3) | 4 | 1 | 1 | IF0S/ADR | Bulk | PR | U | Poor | 3 | 53 |
| RM | – | 4 | 1 | 1 | CB/CY/I/A | MRD | PR | U | Poor | 4 | 46 |
| MG | 26.0 (1.0) | 4 | 1 | 1 | CB/CY/I/A | MRD | PD | E | Poor | 3 | 53 |
| SH | 5.4 (0.1) | 4 | 1 | 1 | CB/CY/I/A | MRD | PR | S | MOD | 4 | 42 |
| EB | 54.1 (1.6) | 4 | 1 | 1 | CB/CY/I/A | MRD | PD | S | MOD | 3 | 61 |
| IF | 31.0 (0.6) | 4 | 1 | 1 | CB | MRD | PD | M | MOD | 3 | 46 |
| LT | 10.34 (0.2) | 4 | 1 | 1 | M | Bulk | PD | U | Poor | 3 | 71 |
| RW | 427.0 (8.5) | 4 | 1 | 1 | CB/CY/I/A | Bulk | PR | S | Poor | 3 | 55 |
| SD | 117.0 (8.2) | 4 | 2 | 1 | CB/CY/I/A | MRD | PR | S | MOD | 3 | 65 |
| MM | 205.0 (8.2) | 4 | 1 | 1 | CIS | MRD | Static | E | MOD | 3 | 64 |
| SW | 68.0 (2.0) | 4 | 1 | 1 | CB/CY/I/A | Bulk | PR | S | Poor | 3 | 66 |
| KW | 97.0 (3.6) | 4 | 1 | 1 | CB/CY/I/A | MRD | PD | E | Poor | 3 | 56 |
| EC | 56.0 (1.1) | 4 | 1 | 1 | CB | Bulk | PD | S | MOD | 3 | 40 |
| PH | 91.0 (2.7) | 4 | 1 | 1 | CB/CY/I/A | MRD | PR | E | Poor | 3 | 62 |
| DG | 137.0 (4.1) | 4 | 2 | 1 | CB | MRD | PD | U | Poor | 3 | 66 |
| MK | 72.0 (1.8) | 4 | 3 | 1 | CB/CY/I/A | Bulk | PR | S | Poor | 3 | 59 |
| DR | 78.0 (2.0) | 4 | 1 | 1 | M | MRD | PD | S | Poor | 3 | 72 |
| MM | 107.00 (3.3) | 4 | 1 | 1 | CB/CY/I/A | Bulk | Static | M | MOD | 2 | 60 |
| DM | 84.0 (3.8) | 4 | 1 | 1 | CB/CY/I/A | MRD | PD | E | Poor | 3 | 42 |
| GA | 92.0 (3.1) | 4 | 1 | 1 | CB | MRD | PD | S | Poor | 3 | 59 |
| PS | 122.0 (4.7) | 4 | 1 | 1 | CB/CY/I/A | Bulk | Static | E | Poor | 3 | 48 |

GSH level - nmoles/mg/protein (mean ± s.d.). GST activity - nmoles conjugate/min/mg protein (mean ± s.d.). Isoenzyme Distribution - % of cells staining positive. 1 - no cells, 2 - few isolated cells, 3- 20% cells, 4 - 20% of cells. Treatment - Drugs used: CB - carboplatin, CY - Cyclophosphamide, A - adriamycin, I - ifosfamide, M - melphelan. Response to treatment: CR - complete response, PR - partial response (> 50% reduction in tumour volume), Static - no change or < 50% reduction. PD - progressive disease. Residual disease at laparotomy: MRD - minimal residual disease less than 2 cm in maximum diameter. Bulk - greater than 2 cm in maximum diameter. Histology: U - unclassifed, E - endemetroid, M - mucinous, S - serous. Differentiation state: poor, moderate, well. Stage-Figo classification.

**Figure 1** **a** Demonstrates the predominantly intracytoplasmic staining found in the majority of tumour cells when stained with antibody to the acidic form of GST. This section was taken from a 28 year old patient with a stage III serous cystadenocarcinoma at first look surgery prior to chemotherapy. **b** Demonstrates a section from the same patient stained for endogenous peroxidase activity. **c** Demonstrates a normal ovary stained with the basic form of GST.

**Table II** GST isoenzyme distribution in tumour, normal ovary and peritoneum

| Proportion of cells expressing GST isoenzymes | Number of samples: Ovary Normal | Ovary 1st Look | Ovary 2nd Look | Ovary Fisher's Exact | Peritoneum Normal | Peritoneum 1st Look | Peritoneum 2nd Look | Peritoneum Fisher's Exact |
|---|---|---|---|---|---|---|---|---|
| *Acidic isoenzymes* | | | | | | | | |
| No cells | | | | | 1 | 3 | 1 | |
| Few isolated | | | | $P = 1.000$ | 8 | 16 | 7 | $P = 0.280$ |
| $<20\%$ | | | | | | | 1 | |
| $> = 20\%$ | 15 | 34 | 21 | | | | 2 | |
| *Basic isoenzymes* | | | | | | | | |
| No cells | | 27 | 18 | $P<0.001$ | 3 | 8 | 4 | |
| Few isolated | | 7 | 2 | $P<0.001$ Normal *vs* 1st Look | 6 | 11 | 6 | |
| | | | | | | | 1 | |
| $<20\%$ | 6 | 2 | 1 | Normal *vs* 2nd Look $P = 0.755$ | | | | $P = 0.819$ |
| $> = 20\%$ | 9 | | | 1st *vs* 2nd Look | | | | |
| *Neutral isoenzymes* | | | | | | | | |
| No cells | 15 | 32 | 21 | | 7 | 12 | 9 | |
| Few isolated | | 4 | | $P = 0.177$ | 2 | 7 | 2 | $P = 0.586$ |
| $<20\%$ | | | | | | | | |
| $> = 20\%$ | | | | | | | | |

**Table III** GST activity in ovarian tumour, normal ovary and peritoneum

| *Tissue* | *No of samples* | *Mean* | *Median* | *Range* | *St. Dev.* |
|---|---|---|---|---|---|
| Normal ovary | 15 | 30.00 | 27.00 | 10.0–66.00 | 21.50 |
| 1st look | 33 | 121.95 | 82.10 | 2.21–652.90 | 153.33 |
| 2nd look | 20 | 94.84 | 81.00 | 5.42–427.00 | 91.90 |
| Peritoneum | 10 | 11.88 | 8.00 | 1.90–30.00 | 10.29 |
| Peritoneum (at 1st look) | 19 | 19.15 | 17.00 | 2.00–45.00 | 13.95 |
| Peritoneum (at 2nd look) | 8 | 20.06 | 12.09 | .08–49.00 | 20.56 |

tion of each of these individual changes to the overall decrease in sensitivity seen in the tumour cells.

Tumours are heterogeneous and also may show a wide range of enzyme activities. Any study which uses tumour homogenates can only give an average value for the tumour. Obviously this cannot give any information about the existence of sub-populations of tumour cells with altered drug sensitivities. Similarly the contribution from normal cells, dying cells, and connective tissue within the tumour cannot be accurately measured. Any interpretation of the GST activity reported in this study must take these limitations into account. The use of antibodies on tissue sections followed by microscopical analysis allows expression of the GST isoenzymes to be observed even in sub-populations of cells within the tumour. However this is a qualitative method, and it would be informative to quantify the expression of each isoenzyme by western blotting. This would be particularly useful for a better characterisation of the apparent decrease in expression of the basic isoform observed in tumours.

This study compares two populations - tumours taken either before or following extensive chemotherapy. The treatment received by these ladies is, generally, a combination of platimum drugs, doxorubicin hydrochloride, and an alkylating agent. Hence the tumour may exhibit resistance to one or all the drugs used. Therefore it is important to try to study as wide a range of proven resistance mechanisms as possible. Glutathione and the glutathione S-transferases have been implicated in resistance to the platinum drugs, doxorubicin hydrochloride, and alkylating agents. These enzymes may therefore be considered as a potential mechanism which may be involved against the drug cocktail used to treat ovarian tumours.

The work of Lewis *et al.* (1988) showed increased GST and GSH in two ovarian cell lines derived from a patient during a course of therapy. This paper also described differences between cells in logarithmic growth compared to confluent cultures. This difference may be important as a determinant of response, as the ovarian tumours are composed mainly of non-cycling cells. However the production of cell lines from tumour biopsy material imposes selective pressures which may result in the cloning of non-representative cell lines.

A study of this type has two major drawbacks. Firstly, it would be better to obtain sequential biopsies from the same patient before and after therapy. A study of this design is at present underway. A comparison of populations (pre-chemotherapy tumours, and normal ovaries) such as that made in this study may not reveal small alterations in GST activity which occurs in tumours following chemotherapy. However no large changes were observed. Computer simulations on the data showed that statistical significance would have been attained if the post-chemotherapy tumours had approximately double the GST activity of the pre-chemotherapy tumours. This increase is less than that reported for many drug resistant cell lines (Misty *et al.*, 1991). Similarly no alterations in isoenzyme distribution between pre- and post-chemotherapy tumours was seen.

Secondly, as already discussed the GST activities represent average values for all cells within the tumour. Evidence for tumour heterogeneity was seen in the distribution of isoenzyme expression with a tumour. However adjacent biopsies from the same tumour or normal ovary showed no significant differences in GST activity. Therefore we have confidence that our measurement of GST in biopsies gives a measure of the average value within the tumour.

In summary these data show that tumours show elevated of glutathione S-transferase activity when compared with normal ovaries. No significant difference was seen betwen tumours excised before or after chemotherapy. These data do not support the hypothesis that large changes in glutathione S-transferase activity are a major determinant of resistance to chemotherapy in ovarian tumours. The possibility still exists that sub-populations of resistant cells exist within the tumour. Similarly if chemotherapy induced only small changes a study of this type may have difficulty in identifying these in a tissue which shows a wide range of intrinsic GST activities. These questions will be better answered by a study using sequential biopsies obtained in the same patient before and after chemotherapy.

This work was supported by the Cancer Research Campaign.

### References

FUJIWARA, Y., SUGIMOTO, Y., KASAHARA, K., BUNGO, M., YAMAKIDO, M., TEW, K.D. & SAIJO, N. (1990). Determinants of drug response in a cisplatin-resistant human lung cancer cell line. *Jpn. J. Cancer Res.*, **81**, 527–535.

GURNEY, H., CROWTHER, D., ANDERSON, H., MURPHY, D., PRENDIVILLE, J., RANSON, M., MAYOR, P., SWINDELL, R., BUCKLEY, C.H. & TINDALL, V.R. (1990). Five year follow-up and dose delivery analysis of cisplatin, iproplatin or carboplatin in combination with cyclophosphamide in advanced ovarian cancer. *Ann. Oncol.*, **1**, 427–433.

HABIG, W.H., PABST, M.J. & JAKOBY, W.B. (1974). Glutathione S-transferases. The first step in mercapturic acid formation. *J. Biol. Chem.*, **249**, 7130–7139.

HALL, A., FOSTER, S., PROCTOR, S.J. & CATTAN, A.R. (1990). Purification and characterization of a pi class glutathione S-transferase from human leukemic cells. *Br. J. Haematol.*, **76**, 494–500.

LEWIS, A.D., HEYES, J.D. & WOLF, C.R. (1988). Glutathione and glutathione dependent enzymes in ovarian adenocarcinoma cell lines derived from a patient before and after the onset of drug resistance: intrinsic differences and cell cycle effects. *Carcinogenesis*, **9**, 1283–1287.

MISTY, P., KELLAND, L.R., ABEL, G., SIODHAR, S. & HARRAP, K.R. (1991). The relationships between glutathione, glutathione S-transferase and cytotoxicity of platinum drugs and melphelan in eight human ovarian carcinoma cell lines. *Br. J. Cancer*, **64**, 215–220.

McGOWN, A.T. & FOX, B.W. (1986). A proposed mechanism of resistance to cyclophosphamide and phosphoramide mustard in a Yoshida cell line *in vitro*. *Cancer Chemother. Pharmacol.*, **17**, 223–226.

NAKAGAWA, K., SAIJO, N., TSICJODA, S., SAKAI, M., TSUROKAWA, Y., YOKOTA, J., MURAMATSU, M., SATO, K., TERADA, M. & TEW, K.D. (1990). Glutathione S-transferase pi as a determinant of drug resistance in transfectant cell lines. *J. Biol. Chem.*, **265**, 4296–4301.

NASH, J.D. & YOUNG, R.C. (1988). Gynecological malignancies. In *Cancer Chemotherapy and Biological Response Modifiers*. Pinedo, H.M., Londo, D.L. & Chabner, B.A. (eds), vol. 10, Amsterdam: Elsevier Science 291–312.

OZOLS, R.F. & YOUNG, R.C. (1984). Chemotherapy of ovarian cancer. *Semin Oncol.*, **11**, 251–263.

RANDALL, B.J., ANGUS, B., AKIBA, R., HALL, A., CATLAN, A.R., PROCTOR, S.J., JONES, R.A. & HORNE, C.H.W. (1990). Glutathione S-transferase (placental) as a marker of transformation in the human cervix uteri: an immunohistochemical study. *Br. J. Cancer*, **62**, 614–618.

ZWELLING, L.A. (1988). Cisplatin and new platinum analogues. In *Cancer Chemotherapy and Biological Response Modifiers*. Pinedo, H.M., Longo, D.L. & Chabner, B.A. (eds), vol. 10, Amsterdam, 64–72.